# Supplementary material for: Whole-body Bacteriophage Distribution Characterized by a Physiologically based Pharmacokinetic Model
Source: bioRxiv. 2025 Feb 6:2025.02.06.636931. Preprint. [Version 1] doi: 10.1101/2025.02.06.636931 (PMC11839030; doi:10.1101/2025.02.06.636931)

bioRxiv preprint doi: https://doi.org/10.1101/2025.02.06.636931; this version posted February 6, 2025. The copyright holder for this preprint (which was not certified by peer review) is the author/funder, who has granted bioRxiv a license to display the preprint in perpetuity. It is made available under aCC-BY 4.0 International license.

SUPPLEMENTAL MATERIALS

Whole-body Phage Distribution Characterized by a Physiologically based Pharmacokinetic Model

Arne Echterhof[1,2], Tejas Dharmaraj[1], Patrick Blankenberg[1], Bobby Targ[1], Paul L. Bollyky[1], Nicholas M. Smith[3] Francis Blankenberg[4]

1) Division of Infectious Diseases and Geographic Medicine, Department of Medicine, Stanford University School of Medicine, Stanford, California, USA.

2) Institute of Medical Microbiology, University Hospital of Muenster, Muenster, Germany

3) Division of Clinical and Translational Therapeutics, School of Pharmacy & Pharmaceutical Sciences, University at Buffalo, Buffalo, New York, USA.
4) Division of Pediatric Radiology and Nuclear Medicine

bioRxiv preprint doi: https://doi.org/10.1101/2025.02.06.636931; this version posted February 6, 2025. The copyright holder for this preprint (which was not certified by peer review) is the author/funder, who has granted bioRxiv a license to display the preprint in perpetuity. It is made available under aCC-BY 4.0 International license.

Table S1: PBPK Model Parameters

Parameter	Definition	Units	Mouse	Rat	Human
CO	Cardiac output	L/h/kg	16.5	15	16.5
BW	Body weight	Kg	0.02	0.25	70
Fr_Q_Brn	Fraction of CO to Brain	-	0.033	0.02	0.114
Fr_Q_Lvr	Fraction of CO to Liver	-	0.021	0.021	0.046
Fr_Q_Spn	Fraction of CO to Spleen	-	0.011	0.0085	0.01375
Fr_Q_SI	Fraction of CO to Sm. Intest.	-	0.105	0.104	0.13575
Fr_Q_LI	Fraction of CO to Lg. Intest.	-	0.0175	0.036	0.022625
Fr_Q_Kid	Fraction of CO to Kidney	-	0.091	0.141	0.175
Fr_Q_Msc	Fraction of CO to muscle	-	0.159	0.278	0.191
Fr_Q_Ski	Fraction of CO to Skin	-	0.058	0.058	0.058
Fr_Q_Bon	Fraction of CO to Bone	-	0.0267	0.122	0.042
Fr_Q_Sto	Fraction of CO to Stomach	-	0.017625	0.013	0.022625
Fr_V_Lun	Fraction of BW as Lung Weight	-	0.0073	0.005	0.008
Fr_V_Brn	Fraction of BW as Brain	-	0.0165	0.00057	0.02
Fr_V_Lvr	Fraction of BW as Liver	-	0.0549	0.0366	0.0257
Fr_V_Spn	Fraction of BW as Lung	-	0.005	0.002	0.00257
Fr_V_SI	Fraction of BW as Sm. Intest	-	0.0253	0.014	0.0091
Fr_V_LI	Fraction of BW as Lg. Intest	-	0.0109	0.0084	0.0053
Fr_V_Kid	Fraction of BW as Kidney	-	0.0167	0.0073	0.0044
Fr_V_Msc	Fraction of BW as Muscle	-	0.384	0.4043	0.4
Fr_V_Ski	Fraction of BW as Skin	-	0.1653	0.1903	0.0371
Fr_V_Mar	Fraction of BW as Bone	-	0.03	0.02	0.02
Fr_V_Sto	Fraction of BW as Stomach	-	0.006	0.0046	0.0021
Fr_V_Pla	Fraction of BW as Blood	-	0.029029	0.074	0.079
Fr_V_Lun_Bld	Fraction of Lung that is Blood	-	0.5	0.36	0.3867
Fr_V_Brn_Bld	Fraction of Brain that is Blood	-	0.03	0.03	0.04
Fr_V_Lvr_Bld	Fraction of Liver that is Blood	-	0.31	0.21	0.11
Fr_V_Spn_Bld	Fraction of Spleen that is Blood	-	0.17	0.22	0.3
Fr_V_SI_Bld	Fraction of Sm. Intest. that is Blood	-	0.24	0.3	0.3
Fr_V_LI_Bld	Fraction of Lg. Intest. that is Blood	-	0.24	0.3	0.3
Fr_V_Kid_Bld	Fraction of Kidney that is blood	-	0.24	0.16	0.36
Fr_V_Msc_Bld	Fraction of Muscle that is blood	-	0.04	0.04	0.01
Fr_V_Ski_Bld	Fraction of Skin that is blood	-	0.03	0.02	0.08

bioRxiv preprint doi: https://doi.org/10.1101/2025.02.06.636931; this version posted February 6, 2025. The copyright holder for this preprint (which was not certified by peer review) is the author/funder, who has granted bioRxiv a license to display the preprint in perpetuity. It is made available under aCC-BY 4.0 International license.

Fr_V_Crs_Bld	Fraction of Carcass that is blood	-	0.04	0.04	0.01
Fr_V_Bon_Bld	Fraction of Bone that is blood	-	0.1	0.04	0.04
Fr_V_Sto_Bld	Fraction of stomach that is blood	-	0.24	0.3	0.3
Phagocytes_Pla	Phagocytes in Blood	cells/g	180000	14300	4170000
Phagocytes_Lun	Phagocytes in Lung	cells/g	1710000	9210000	24800000
Phagocytes_Brn	Phagocytes in Brain	cells/g	180000	390000	8400000
Phagocytes_Lvr	Phagocytes in Liver	cells/g	1710000	2.70E+07	21800000
Phagocytes_Spn	Phagocytes in Spleen	cells/g	1710000	2.28E+08	1.18E+08
Phagocytes_SI	Phagocytes in Sm. Intest.	cells/g	180000	60000	1710000
Phagocytes_LI	Phagocytes in Lg. Intest	cells/g	180000	15000	11200000
Phagocytes_Kid	Phagocytes in Kidney	cells/g	180000	390000	2210000
Phagocytes_Crs	Phagocytes in Carcass	cells/g	180000	6350000	2630000
Phagocytes_Msc	Phagocytes in Muscle	cells/g	180000	40000	773000
Phagocytes_Ski	Phagocytes in Skin	cells/g	180000	40000	3910000
Phagocytes_Bon	Phagocytes in Bone	cells/g	180000	14900000	45300000
Phagocytes_Sto	Phagocytes in Stomach	cells/g	180000	6350000	936000

bioRxiv preprint doi: https://doi.org/10.1101/2025.02.06.636931; this version posted February 6, 2025. The copyright holder for this preprint (which was not certified by peer review) is the author/funder, who has granted bioRxiv a license to display the preprint in perpetuity. It is made available under aCC-BY 4.0 International license.

Equations

Lung (Lun)

$$\frac{dC_{Lun,V}}{dt} \cdot V_{Lun,V} = Q_{Lun} \cdot \left(C_a(t) - C_{Lun,V}(t)\right) - P_S \cdot Q_{Lun} \cdot \left(C_{Lun,V}(t) - \frac{C_{Lun,int}(t)}{K_{p,LunKid}}\right)$$

$$\frac{dC_{Lun,int}}{dt} \cdot V_{Lun,int} = P_S \cdot Q_{Lun} \cdot \left(C_{Lun,V}(t) - \frac{C_{Lun,int}(t)}{K_{p,LunKid}}\right) - k_{Lun,up}(t) \cdot C_{Lun,int}(t) \cdot V_{Lun,int} + k_{rel} \cdot A_{Lun,res}(t)$$

$$\frac{dA_{Lun,res}}{dt} = k_{Lun,up}(t) \cdot C_{Lun,int}(t) \cdot V_{Lun,int} - k_{rel} \cdot A_{Lun,res}(t) - k_{deg} \cdot A_{Lun,res}(t)$$

$$k_{Lun,up}(t) = k_{up,max} \cdot \left(1 - \frac{A_{Lun,res}(t)}{A_{Lun,max}}\right)$$

$$A_{Lun,max} = M_{Lun} \cdot V_{Lun} \cdot \left(\frac{A_{RES}}{10^5}\right)$$

Muscle (Msc)

$$\frac{dC_{Msc,V}}{dt} \cdot V_{Msc,V} = Q_{Msc} \cdot \left(C_a(t) - C_{Msc,V}(t)\right) - P_S \cdot Q_{Msc} \cdot \left(C_{Msc,V}(t) - \frac{C_{Msc,int}(t)}{K_{p,Crs}}\right)$$

$$\frac{dC_{Msc,int}}{dt} \cdot V_{Msc,int} = P_S \cdot Q_{Msc} \cdot \left(C_{Msc,V}(t) - \frac{C_{Msc,int}(t)}{K_{p,Crs}}\right) - k_{Msc,up}(t) \cdot C_{Msc,int}(t) \cdot V_{Msc,int} + k_{rel} \cdot A_{Msc,res}(t)$$

$$\frac{dA_{Msc,res}}{dt} = k_{Msc,up}(t) \cdot C_{Msc,int}(t) \cdot V_{Msc,int} - k_{rel} \cdot A_{Msc,res}(t) - k_{deg} \cdot A_{Msc,res}(t)$$

$$k_{Msc,up}(t) = k_{up,max} \cdot \left(1 - \frac{A_{MSc,res}(t)}{A_{Msc,max}}\right)$$

$$A_{Msc,max} = M_{Msc} \cdot V_{Msc} \cdot \left(\frac{A_{RES}}{10^5}\right)$$

Bone (Bon)

$$\frac{dC_{Bon,V}}{dt} \cdot V_{Bon,V} = Q_{Bon} \cdot \left(C_a(t) - C_{Bon,V}(t)\right) - P_S \cdot Q_{Bon} \cdot \left(C_{Bon,V}(t) - \frac{C_{Bon,int}(t)}{K_{p,Crs}}\right)$$

$$\frac{dC_{Bon,int}}{dt} \cdot V_{Bon,int} = P_S \cdot Q_{Bon} \cdot \left(C_{Bon,V}(t) - \frac{C_{Bon,int}(t)}{K_{p,Crs}}\right) - k_{Bon,up}(t) \cdot C_{Bon,int}(t) \cdot V_{Bon,int} + k_{rel} \cdot A_{Bon,res}(t)$$

$$\frac{dA_{Bon,res}}{dt} = k_{Bon,up}(t) \cdot C_{Bon,int}(t) \cdot V_{Bon,int} - k_{rel} \cdot A_{Bon,res}(t) - k_{deg} \cdot A_{Bon,res}(t)$$

$$k_{Bon,up}(t) = k_{up,max} \cdot \left(1 - \frac{A_{Bon,res}(t)}{A_{Bon,max}}\right)$$

$$A_{Bon,max} = M_{Bon} \cdot V_{Bon} \cdot \left(\frac{A_{RES}}{10^5}\right)$$

Liver (Lvr)

bioRxiv preprint doi: https://doi.org/10.1101/2025.02.06.636931; this version posted February 6, 2025. The copyright holder for this preprint (which was not certified by peer review) is the author/funder, who has granted bioRxiv a license to display the preprint in perpetuity. It is made available under aCC-BY 4.0 International license.

$$\frac{dC_{Lvr,V}}{dt} \cdot V_{Lvr,V} = Q_{Lvr} \cdot C_a(t) - \left(Q_{Lvr} + Q_{Hpv}\right) \cdot C_{Lvr,V}(t) + Q_{Spn} \cdot C_{Spn,V}(t) + Q_{SI} \cdot C_{SI,V}(t) + Q_{LI} \cdot C_{LI,V}(t) + Q_{Sto} \cdot C_{Sto,V}(t) - P_S \cdot Q_{Lvr} \cdot \left(C_{Lvr,V}(t) - \frac{C_{Lvr,int}(t)}{K_{p,LvrSpnSto}}\right) - CL_{Active} \cdot C_{Lvr,V}(t)$$

$$Q_{Hpv} = Q_{Spn} + Q_{Sto} + Q_{SI} + Q_{LI}$$

$$\frac{dC_{Lvr,int}}{dt} \cdot V_{Lvr,int} = P_S \cdot Q_{Lvr} \cdot \left(C_{Lvr,V}(t) - \frac{C_{Lvr,int}(t)}{K_{p,LvrSpnSto}}\right) - k_{Lvr,up}(t) \cdot C_{Lvr,int}(t) \cdot V_{Lvr,int} + k_{rel} \cdot A_{Lvr,res}(t)$$

$$\frac{dA_{Lvr,res}}{dt} = k_{Lvr,up}(t) \cdot C_{Lvr,int}(t) \cdot V_{Lvr,int} - k_{rel} \cdot A_{Lvr,res}(t) - k_{deg} \cdot A_{Lvr,res}(t)$$

$$k_{Lvr,up}(t) = k_{up,max} \cdot \left(1 - \frac{A_{Lvr,res}(t)}{A_{Lvr,max}}\right)$$

$$A_{Lvr,max} = M_{Lvr} \cdot V_{Lvr} \cdot \left(\frac{A_{RES}}{10^5}\right)$$

Stomach (Sto)

$$\frac{dC_{Sto,V}}{dt} \cdot V_{Sto,V} = Q_{Sto} \cdot \left(C_a(t) - C_{Sto,V}(t)\right) - P_S \cdot Q_{Sto} \cdot \left(C_{Sto,V}(t) - \frac{C_{Sto,int}(t)}{K_{p,LvrSpnSto}}\right) - CL_{Active} \cdot C_{Sto,V}(t)$$

$$\frac{dC_{Sto,int}}{dt} \cdot V_{Sto,int} = P_S \cdot Q_{Sto} \cdot \left(C_{Sto,V}(t) - \frac{C_{Sto,int}(t)}{K_{p,LvrSpnSto}}\right) - k_{Sto,up}(t) \cdot C_{Sto,int}(t) \cdot V_{Sto,int} + k_{rel} \cdot A_{Sto,res}(t)$$

$$\frac{dA_{Sto,res}}{dt} = k_{Sto,up}(t) \cdot C_{Sto,int}(t) \cdot V_{Sto,int} - k_{rel} \cdot A_{Sto,res}(t) - k_{deg} \cdot A_{Sto,res}(t)$$

$$k_{Sto,up}(t) = k_{up,max} \cdot \left(1 - \frac{A_{Sto,res}(t)}{A_{Sto,max}}\right)$$

$$A_{Sto,max} = M_{Sto} \cdot V_{Sto} \cdot \left(\frac{A_{RES}}{10^5}\right)$$

Spleen (Spn)

$$\frac{dC_{Spn,V}}{dt} \cdot V_{Spn,V} = Q_{Spn} \cdot \left(C_a(t) - C_{Spn,V}(t)\right) - P_S \cdot Q_{Spn} \cdot \left(C_{Spn,V}(t) - \frac{C_{Spn,int}(t)}{K_{p,LvrSpnSto}}\right)$$

$$\frac{dC_{Spn,int}}{dt} \cdot V_{Spn,int} = P_S \cdot Q_{Spn} \cdot \left(C_{Spn,V}(t) - \frac{C_{Spn,int}(t)}{K_{p,LvrSpnSto}}\right) - k_{Spn,up}(t) \cdot C_{Spn,int}(t) \cdot V_{Spn,int} + k_{rel} \cdot A_{Spn,res}(t)$$

$$\frac{dA_{Spn,res}}{dt} = k_{Spn,up}(t) \cdot C_{Spn,int}(t) \cdot V_{Spn,int} - k_{rel} \cdot A_{Spn,res}(t) - k_{deg} \cdot A_{Spn,res}(t)$$

$$k_{Spn,up}(t) = k_{up,max} \cdot \left(1 - \frac{A_{Spn,res}(t)}{A_{Spn,max}}\right)$$

$$A_{Spn,max} = M_{Spn} \cdot V_{Spn} \cdot \left(\frac{A_{RES}}{10^5}\right)$$

Small Intestine (SI)

bioRxiv preprint doi: https://doi.org/10.1101/2025.02.06.636931; this version posted February 6, 2025. The copyright holder for this preprint (which was not certified by peer review) is the author/funder, who has granted bioRxiv a license to display the preprint in perpetuity. It is made available under aCC-BY 4.0 International license.

$$\frac{dC_{SI,V}}{dt} \cdot V_{SI,V} = Q_{SI} \cdot \left(C_a(t) - C_{SI,V}(t)\right) - P_S \cdot Q_{SI} \cdot \left(C_{SI,V}(t) - \frac{C_{SI,int}(t)}{K_{p,Crs}}\right) - CL_{Active} \cdot C_{SI,V}(t)$$

$$\frac{dC_{SI,int}}{dt} \cdot V_{SI,int} = P_S \cdot Q_{SI} \cdot \left(C_{SI,V}(t) - \frac{C_{SI,int}(t)}{K_{p,Crs}}\right) - k_{SI,up}(t) \cdot C_{SI,int}(t) \cdot V_{SI,int} + k_{rel} \cdot A_{SI,res}(t)$$

$$\frac{dA_{SI,res}}{dt} = k_{SI,up}(t) \cdot C_{SI,int}(t) \cdot V_{SI,int} - k_{rel} \cdot A_{SI,res}(t) - k_{deg} \cdot A_{SI,res}(t)$$

$$k_{Spn,up}(t) = k_{up,max} \cdot \left(1 - \frac{A_{Spn,res}(t)}{A_{Spn,max}}\right)$$

$$A_{Spn,max} = M_{Spn} \cdot V_{Spn} \cdot \left(\frac{A_{RES}}{10^5}\right)$$

Large Intestine (LI)

$$\frac{dC_{LI,V}}{dt} \cdot V_{LI,V} = Q_{LI} \cdot \left(C_a(t) - C_{LI,V}(t)\right) - P_S \cdot Q_{LI} \cdot \left(C_{LI,V}(t) - \frac{C_{LI,int}(t)}{K_{p,Crs}}\right) - CL_{Active} \cdot C_{LI,V}(t)$$

$$\frac{dC_{LI,int}}{dt} \cdot V_{LI,int} = P_S \cdot Q_{LI} \cdot \left(C_{LI,V}(t) - \frac{C_{LI,int}(t)}{K_{p,Crs}}\right) - k_{LI,up}(t) \cdot C_{LI,int}(t) \cdot V_{LI,int} + k_{rel} \cdot A_{LI,res}(t)$$

$$\frac{dA_{LI,res}}{dt} = k_{LI,up}(t) \cdot C_{LI,int}(t) \cdot V_{LI,int} - k_{rel} \cdot A_{LI,res}(t) - k_{deg} \cdot A_{LI,res}(t)$$

$$k_{LI,up}(t) = k_{up,max} \cdot \left(1 - \frac{A_{LI,res}(t)}{A_{LI,max}}\right)$$

$$A_{LI,max} = M_{LI} \cdot V_{LI} \cdot \left(\frac{A_{RES}}{10^5}\right)$$

Brain (Brn)

$$\frac{dC_{Brn,V}}{dt} \cdot V_{Brn,V} = Q_{Brn} \cdot \left(C_a(t) - C_{Brn,V}(t)\right) - P_S \cdot Q_{Brn} \cdot \left(C_{Brn,V}(t) - \frac{C_{Brn,int}(t)}{K_{p,Crs}}\right)$$

$$\frac{dC_{Brn,int}}{dt} \cdot V_{Brn,int} = P_S \cdot Q_{Brn} \cdot \left(C_{Brn,V}(t) - \frac{C_{Brn,int}(t)}{K_{p,Crs}}\right)$$

Skin (Ski)

$$\frac{dC_{Ski,V}}{dt} \cdot V_{Ski,V} = Q_{Brn} \cdot \left(C_a(t) - C_{Ski,V}(t)\right) - P_S \cdot Q_{Ski} \cdot \left(C_{Ski,V}(t) - \frac{C_{Ski,int}(t)}{K_{p,Crs}}\right)$$

$$\frac{dC_{Ski,V}}{dt} \cdot V_{Ski,V} = P_S \cdot Q_{Ski} \cdot \left(C_{Ski,V}(t) - \frac{C_{Ski,int}(t)}{K_{p,Crs}}\right) - k_{Ski,up}(t) \cdot C_{Ski,int}(t) \cdot V_{Ski,int} + k_{rel} \cdot A_{Ski,res}(t)$$

$$\frac{dA_{Ski,res}}{dt} = k_{Ski,up}(t) \cdot C_{Ski,int}(t) \cdot V_{Ski,int} - k_{rel} \cdot A_{LI,res}(t) - k_{deg} \cdot A_{Ski,res}(t)$$

$$k_{Ski,up}(t) = k_{up,max} \cdot \left(1 - \frac{A_{Ski,res}(t)}{A_{Ski,max}}\right)$$

$$A_{Ski,max} = M_{Ski} \cdot V_{Ski} \cdot \left(\frac{A_{RES}}{10^5}\right)$$

Carcass (Crs)

$$\frac{dC_{Crs,V}}{dt} \cdot V_{Crs,V} = Q_{Crs} \cdot \left(C_a(t) - C_{Crs,V}(t)\right) - P_S \cdot Q_{Crs} \cdot \left(C_{Crs,V}(t) - \frac{C_{Crs,int}(t)}{K_{p,Crs}}\right)$$

bioRxiv preprint doi: https://doi.org/10.1101/2025.02.06.636931; this version posted February 6, 2025. The copyright holder for this preprint (which was not certified by peer review) is the author/funder, who has granted bioRxiv a license to display the preprint in perpetuity. It is made available under aCC-BY 4.0 International license.

$$\frac{dC_{Crs,V}}{dt} \cdot V_{Crs,V} = P_S \cdot Q_{Crs} \cdot \left(C_{Crs,V}(t) - \frac{C_{Crs,int}(t)}{K_{p,Crs}}\right) - k_{Crs,up}(t) \cdot C_{Crs,int}(t) \cdot V_{Crs,int} + k_{rel} \cdot A_{Crs,res}(t)$$

$$\frac{dA_{Crs,res}}{dt} = k_{Crs,up}(t) \cdot C_{Crs,int}(t) \cdot V_{Crs,int} - k_{rel} \cdot A_{Crs,res}(t) - k_{deg} \cdot A_{Crs,res}(t)$$

$$k_{Crs,up}(t) = k_{up,max} \cdot \left(1 - \frac{A_{Crs,res}(t)}{A_{Crs,max}}\right)$$

$$A_{Crs,max} = M_{Crs} \cdot V_{Crs} \cdot \left(\frac{A_{RES}}{10^5}\right)$$

Kidney (Kid)

$$\frac{dC_{Kid,V}}{dt} \cdot V_{Kid,V} = Q_{Kid} \cdot \left(C_a(t) - C_{Kid,V}(t)\right) - P_S \cdot Q_{Kid} \cdot \left(C_{Kid,V}(t) - \frac{C_{Kid,int}(t)}{K_{p,LunKid}}\right) - CL_{Active} \cdot C_{Kid,V}(t)$$

$$\frac{dC_{Kid,V}}{dt} \cdot V_{Kid,V} = P_S \cdot Q_{Kid} \cdot \left(C_{Kid,V}(t) - \frac{C_{Kid,int}(t)}{K_{p,LunKid}}\right) - k_{Kid,up}(t) \cdot C_{Kid,int}(t) \cdot V_{Kid,int} + k_{rel} \cdot A_{Kid,res}(t)$$

$$\frac{dA_{Kid,res}}{dt} = k_{Kid,up}(t) \cdot C_{Kid,int}(t) \cdot V_{Kid,int} - k_{rel} \cdot A_{Kid,res}(t) - k_{deg} \cdot A_{Kid,res}(t)$$

$$\frac{dA_{Kid,Urine}}{dt} = CL_{Active} \cdot C_{Kid,V}(t) - k_{Kid,urine} \cdot A_{Kid,Urine}(t)$$

$$k_{Kid,up}(t) = k_{up,max} \cdot \left(1 - \frac{A_{Kid,res}(t)}{A_{Kid,max}}\right)$$

$$A_{Kid,max} = M_{Kid} \cdot V_{Kid} \cdot \left(\frac{A_{RES}}{10^5}\right)$$

Venous

$$\frac{dC_V}{dt} \cdot V_V = Q_{Brn} \cdot C_{Brn,v}(t) + \left(Q_{Lvr} + Q_{Hpv}\right) \cdot C_{Lvr,v}(t) + Q_{Kid} \cdot C_{Kid,v}(t) + Q_{Msc} \cdot C_{Msc,v}(t) + Q_{Ski} \cdot C_{Ski,v}(t) + Q_{Bon} \cdot C_{Bon,v}(t) + Q_{Crs} \cdot C_{Crs,v}(t) - Q_{Lun} \cdot C_{Lun,v}(t) - k_{V,up} \cdot C_V(t) \cdot V_V + k_{rel} \cdot A_{V,res}(t)$$

bioRxiv preprint doi: https://doi.org/10.1101/2025.02.06.636931; this version posted February 6, 2025. The copyright holder for this preprint (which was not certified by peer review) is the author/funder, who has granted bioRxiv a license to display the preprint in perpetuity. It is made available under aCC-BY 4.0 International license.

Figure S1: Observed versus predicted diagnostic plot of Blood measurements.

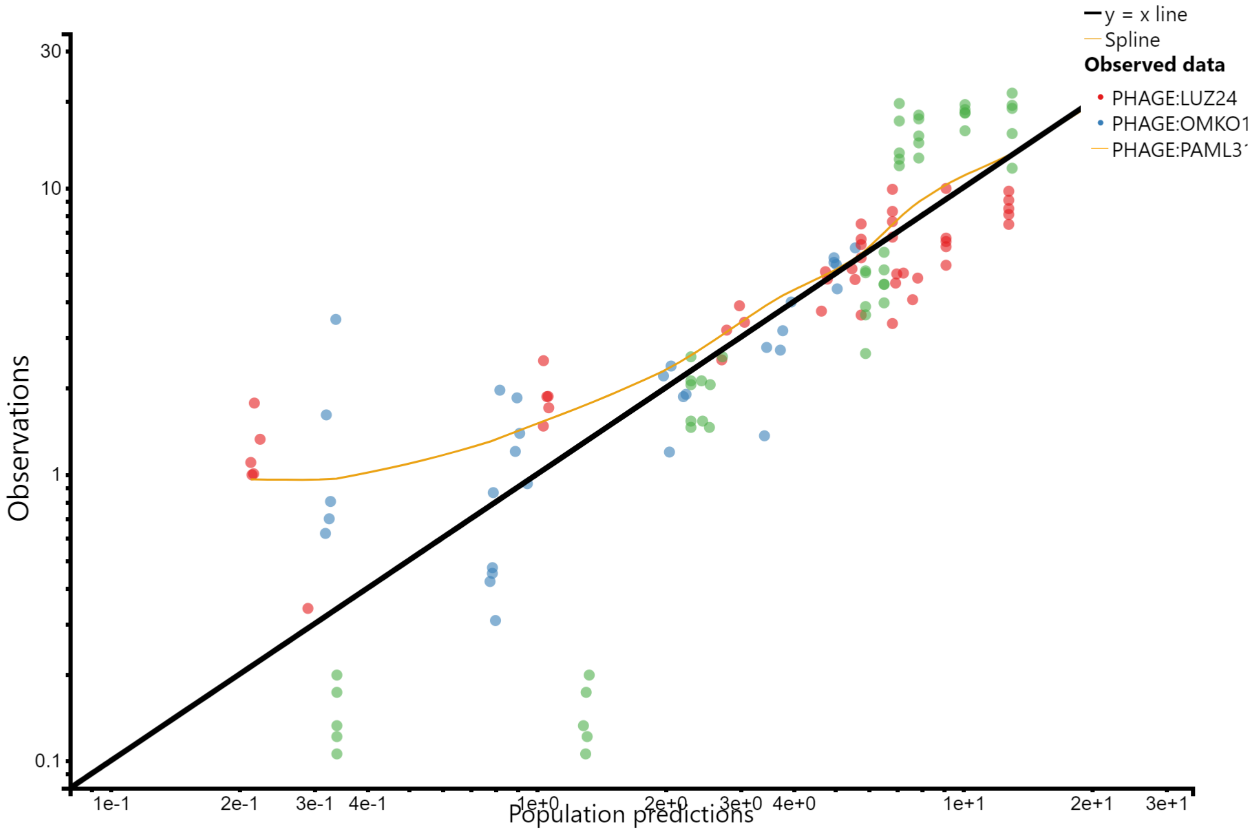

bioRxiv preprint doi: https://doi.org/10.1101/2025.02.06.636931; this version posted February 6, 2025. The copyright holder for this preprint (which was not certified by peer review) is the author/funder, who has granted bioRxiv a license to display the preprint in perpetuity. It is made available under aCC-BY 4.0 International license.

Figure S2: Observed versus predicted diagnostic plot of Bone measurements.

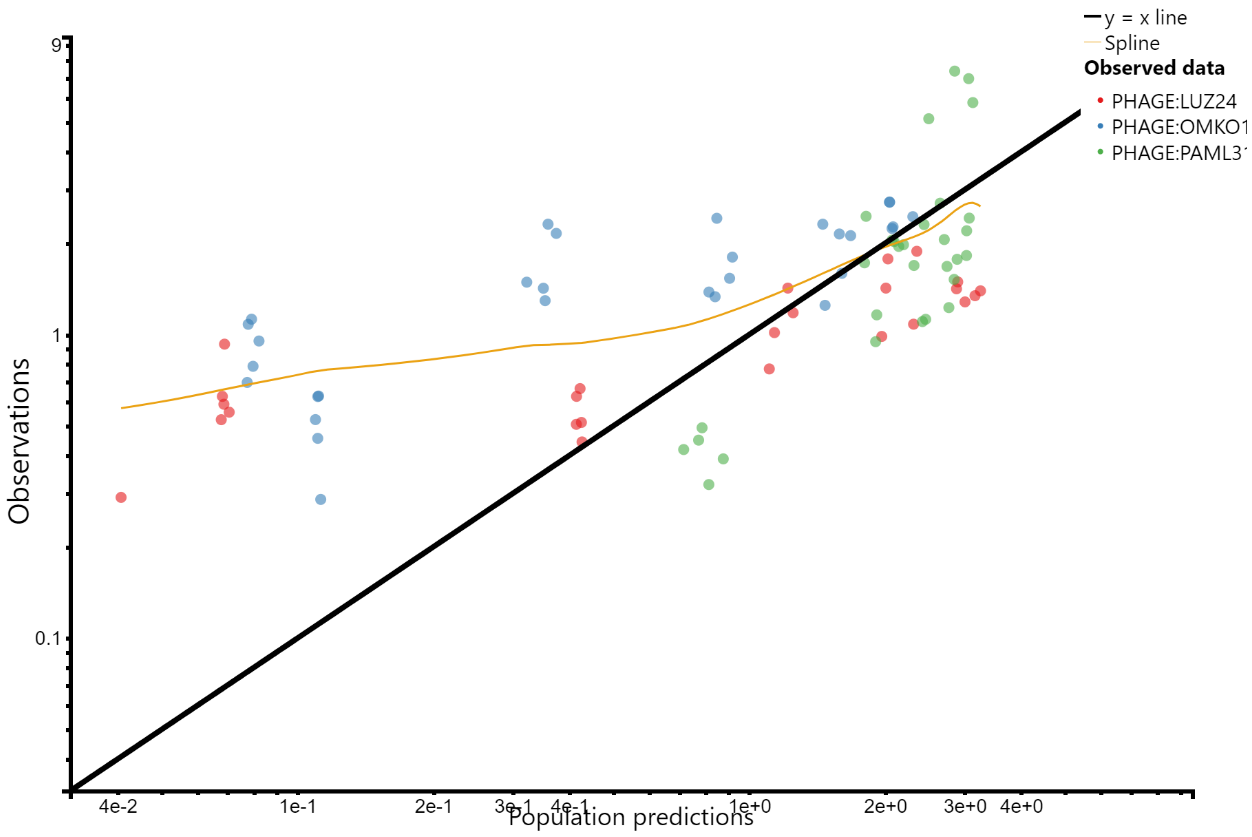

bioRxiv preprint doi: https://doi.org/10.1101/2025.02.06.636931; this version posted February 6, 2025. The copyright holder for this preprint (which was not certified by peer review) is the author/funder, who has granted bioRxiv a license to display the preprint in perpetuity. It is made available under aCC-BY 4.0 International license.

Figure S3: Observed versus predicted diagnostic plot of Kidney measurements.

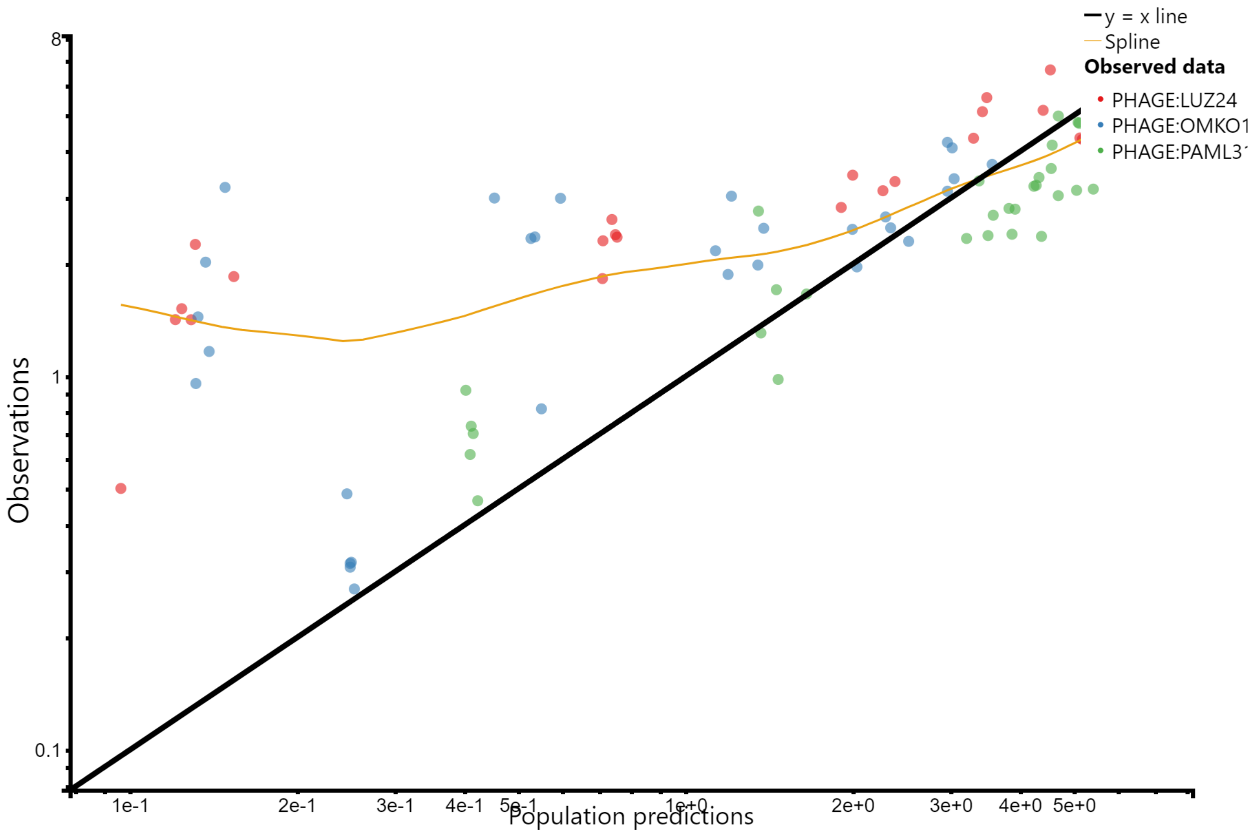

bioRxiv preprint doi: https://doi.org/10.1101/2025.02.06.636931; this version posted February 6, 2025. The copyright holder for this preprint (which was not certified by peer review) is the author/funder, who has granted bioRxiv a license to display the preprint in perpetuity. It is made available under aCC-BY 4.0 International license.

Figure S4: Observed versus predicted diagnostic plot of Lg. Intestines measurements.

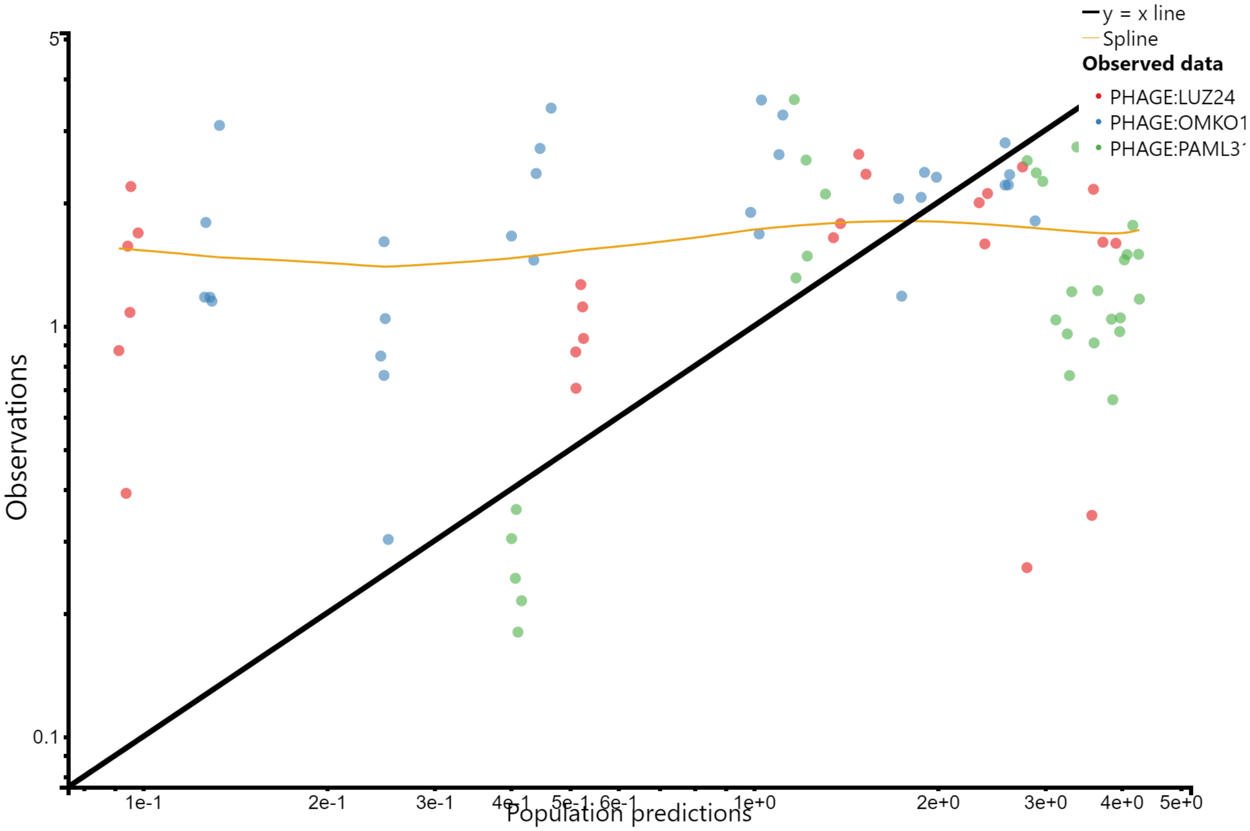

bioRxiv preprint doi: https://doi.org/10.1101/2025.02.06.636931; this version posted February 6, 2025. The copyright holder for this preprint (which was not certified by peer review) is the author/funder, who has granted bioRxiv a license to display the preprint in perpetuity. It is made available under aCC-BY 4.0 International license.

Figure S5: Observed versus predicted diagnostic plot of Liver measurements.

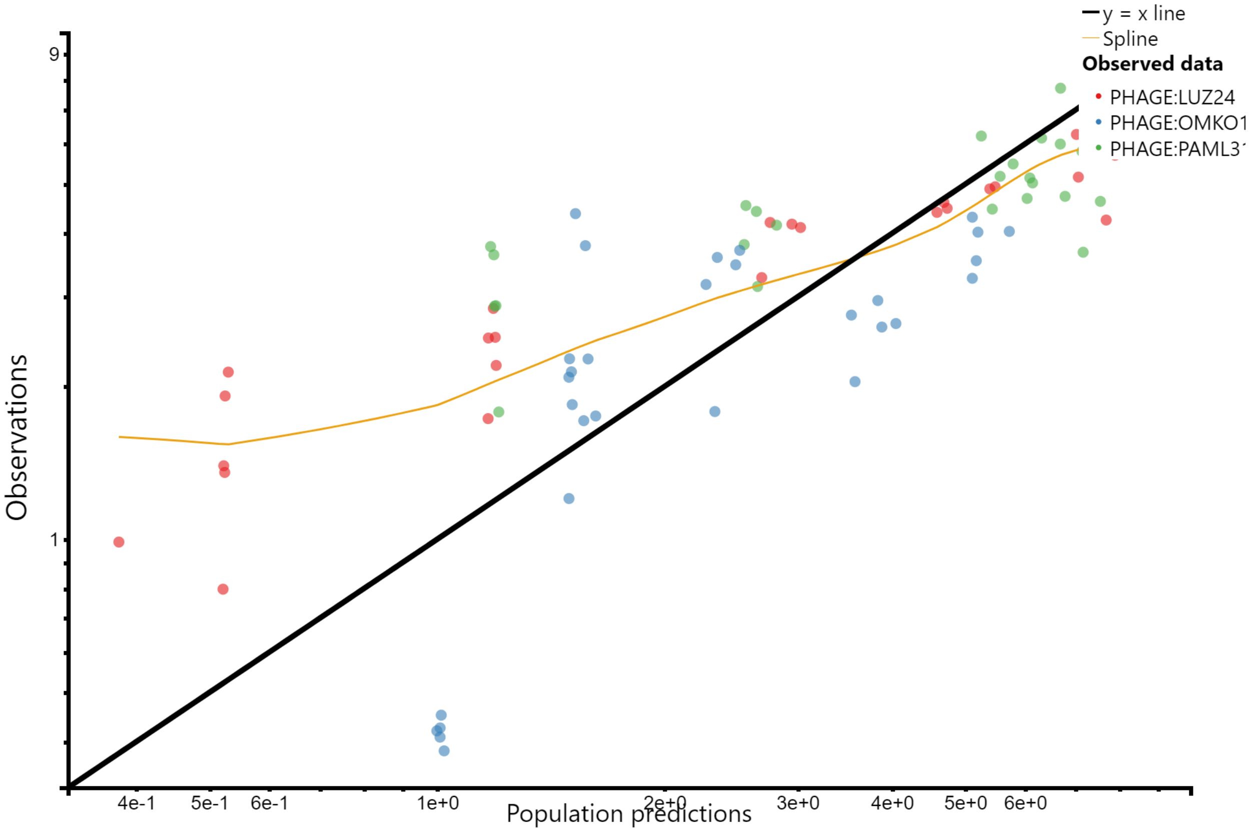

bioRxiv preprint doi: https://doi.org/10.1101/2025.02.06.636931; this version posted February 6, 2025. The copyright holder for this preprint (which was not certified by peer review) is the author/funder, who has granted bioRxiv a license to display the preprint in perpetuity. It is made available under aCC-BY 4.0 International license.

Figure S6: Observed versus predicted diagnostic plot of Lung measurements.

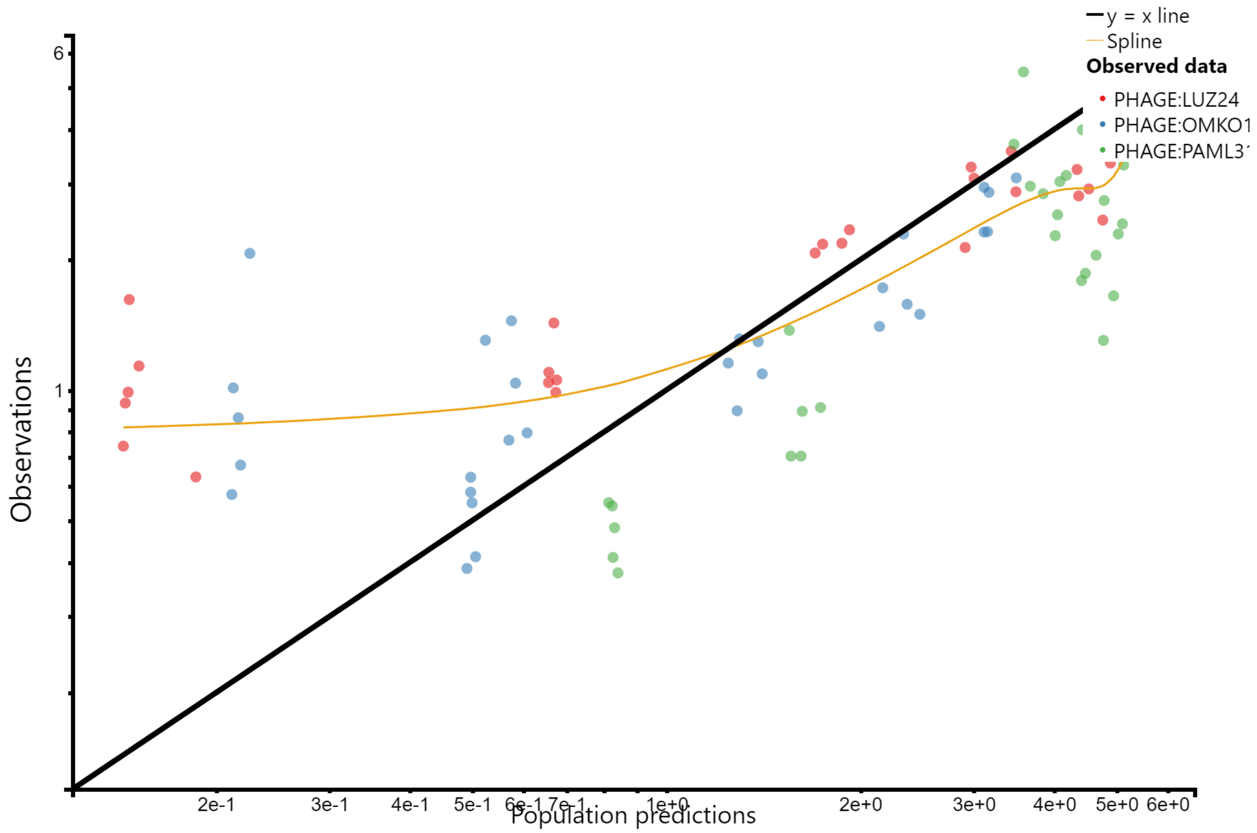

bioRxiv preprint doi: https://doi.org/10.1101/2025.02.06.636931; this version posted February 6, 2025. The copyright holder for this preprint (which was not certified by peer review) is the author/funder, who has granted bioRxiv a license to display the preprint in perpetuity. It is made available under aCC-BY 4.0 International license.

Figure S7: Observed versus predicted diagnostic plot of Muscle measurements.

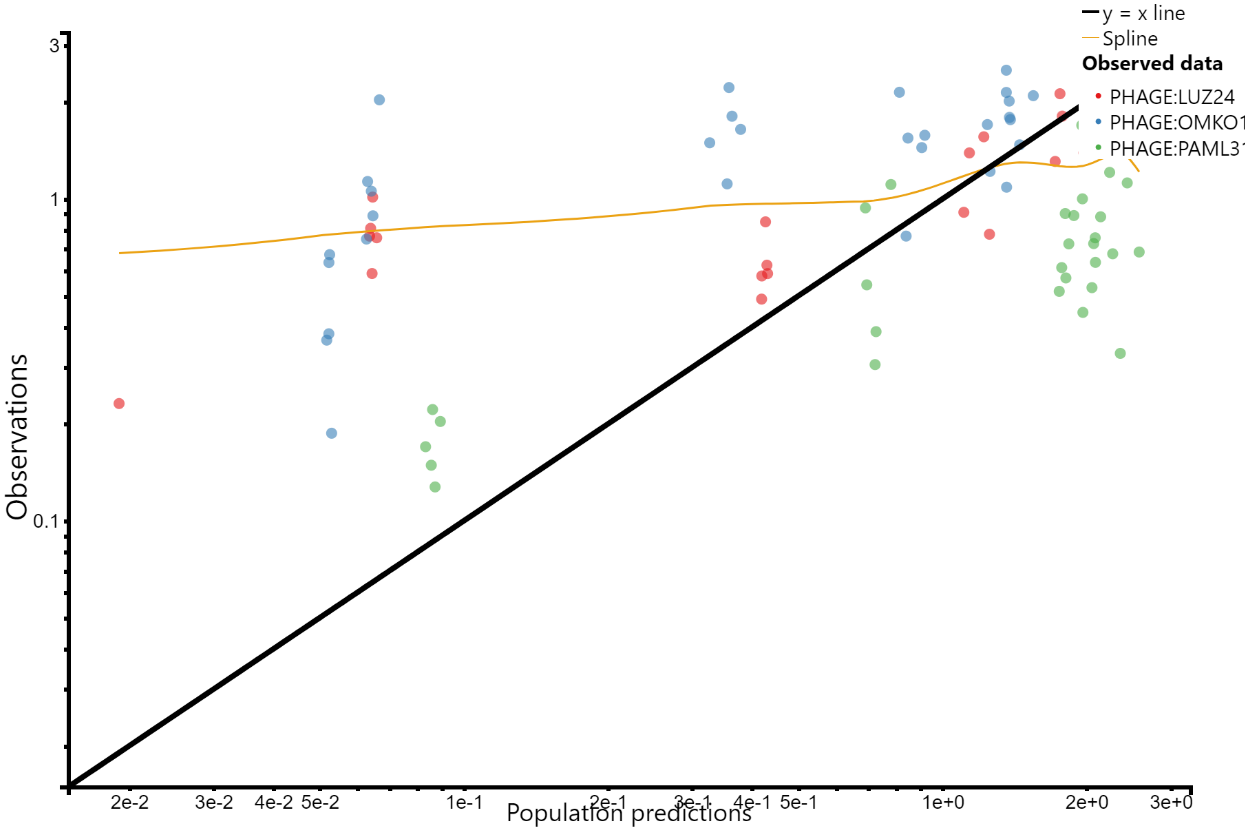

bioRxiv preprint doi: https://doi.org/10.1101/2025.02.06.636931; this version posted February 6, 2025. The copyright holder for this preprint (which was not certified by peer review) is the author/funder, who has granted bioRxiv a license to display the preprint in perpetuity. It is made available under aCC-BY 4.0 International license.

Figure S8: Observed versus predicted diagnostic plot of Sm. Intestines measurements.

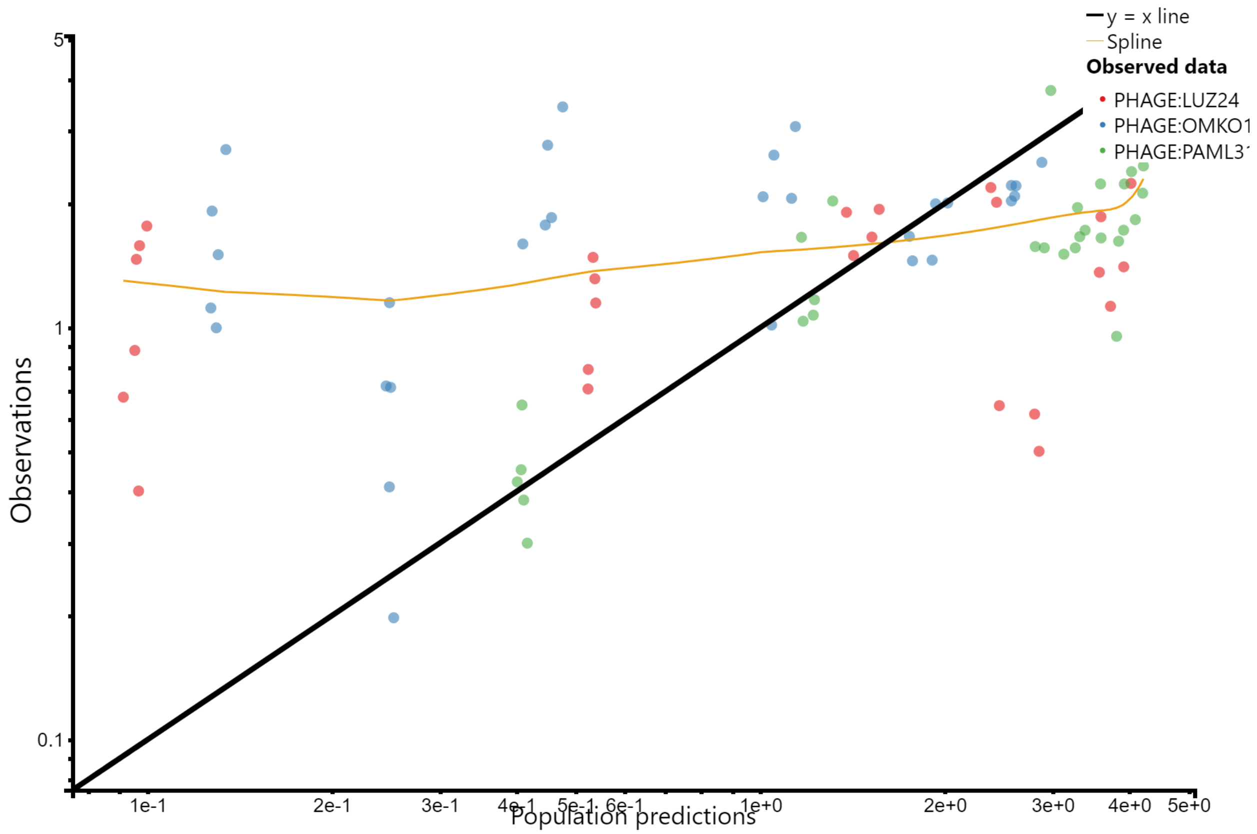

bioRxiv preprint doi: https://doi.org/10.1101/2025.02.06.636931; this version posted February 6, 2025. The copyright holder for this preprint (which was not certified by peer review) is the author/funder, who has granted bioRxiv a license to display the preprint in perpetuity. It is made available under aCC-BY 4.0 International license.

Figure S9: Observed versus predicted diagnostic plot of Spleen measurements.

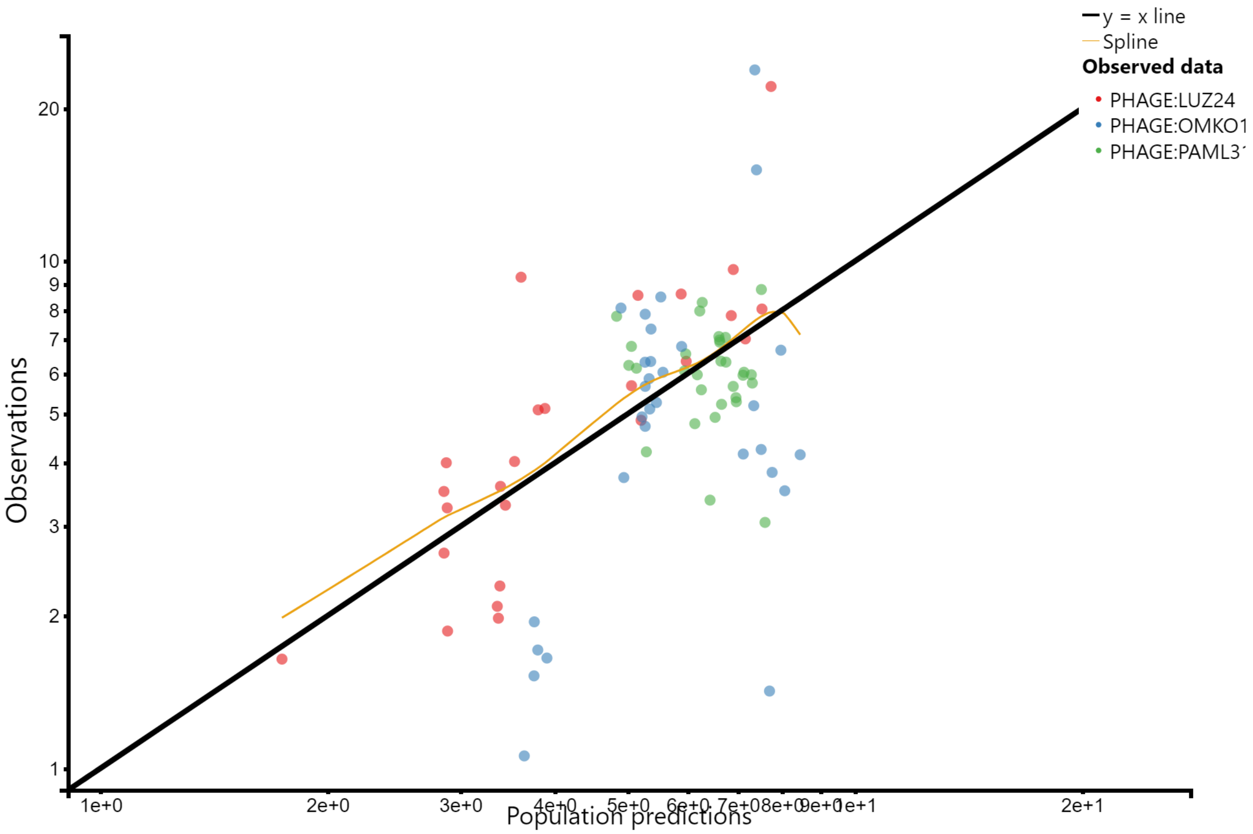

bioRxiv preprint doi: https://doi.org/10.1101/2025.02.06.636931; this version posted February 6, 2025. The copyright holder for this preprint (which was not certified by peer review) is the author/funder, who has granted bioRxiv a license to display the preprint in perpetuity. It is made available under aCC-BY 4.0 International license.

Figure S10: Observed versus predicted diagnostic plot of Stomach measurements.

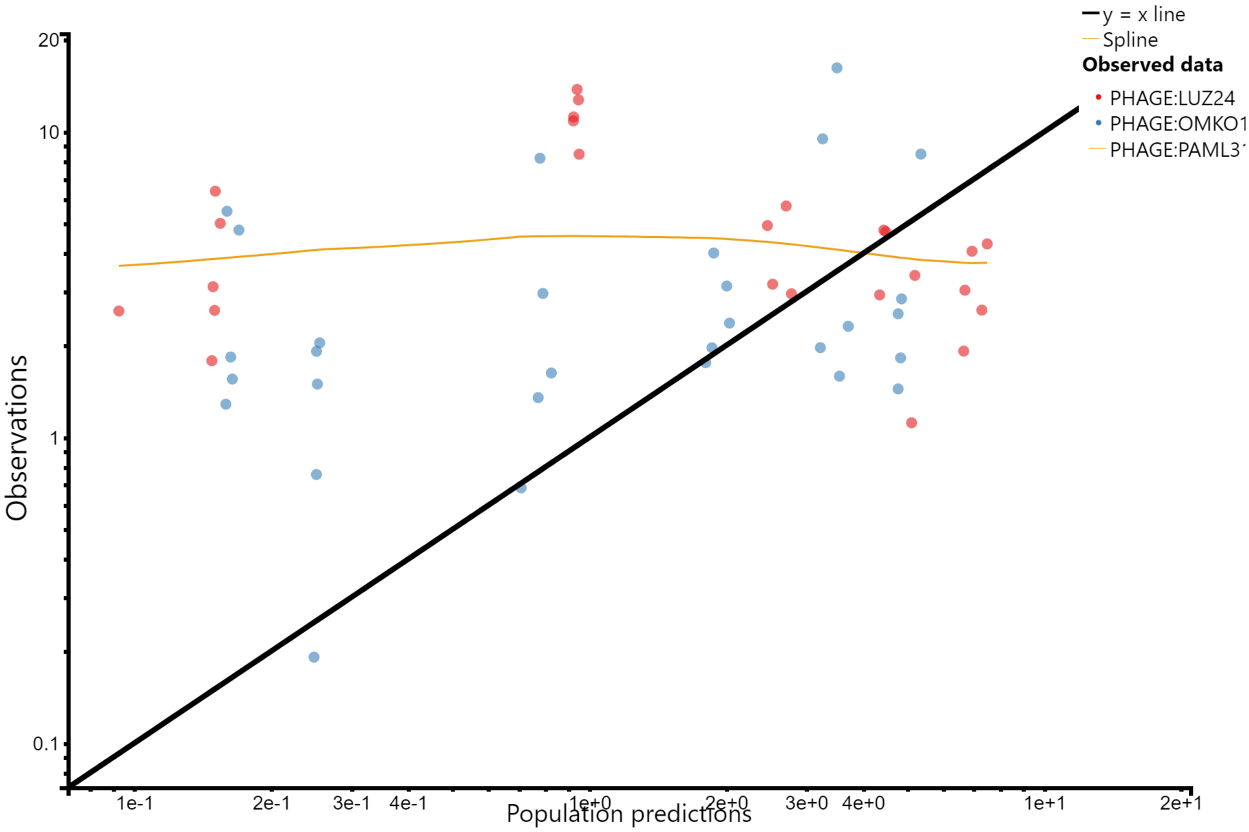

bioRxiv preprint doi: https://doi.org/10.1101/2025.02.06.636931; this version posted February 6, 2025. The copyright holder for this preprint (which was not certified by peer review) is the author/funder, who has granted bioRxiv a license to display the preprint in perpetuity. It is made available under aCC-BY 4.0 International license.

Figure S11: Observed versus predicted diagnostic plot of Stomach Contents measurements.

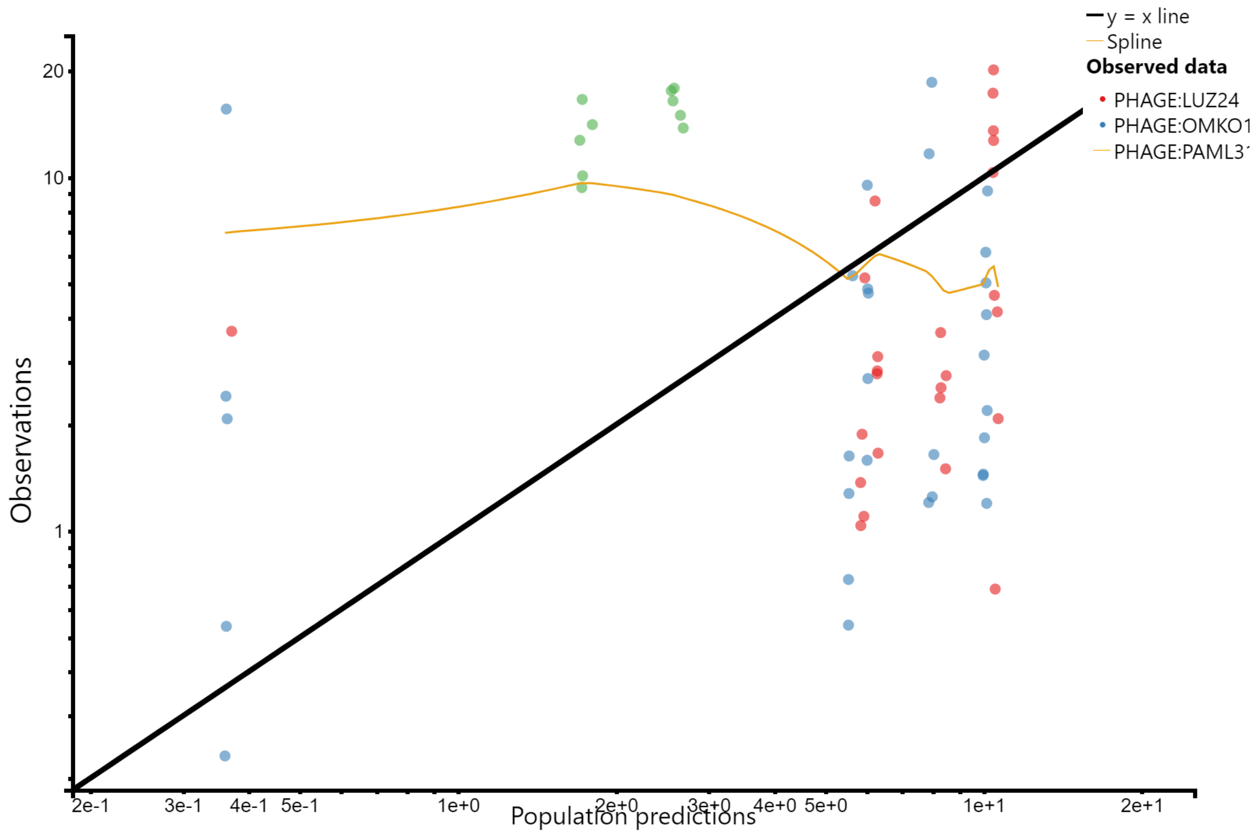

bioRxiv preprint doi: https://doi.org/10.1101/2025.02.06.636931; this version posted February 6, 2025. The copyright holder for this preprint (which was not certified by peer review) is the author/funder, who has granted bioRxiv a license to display the preprint in perpetuity. It is made available under aCC-BY 4.0 International license.

Figure S12: Observed versus predicted diagnostic plot of Urine measurements.

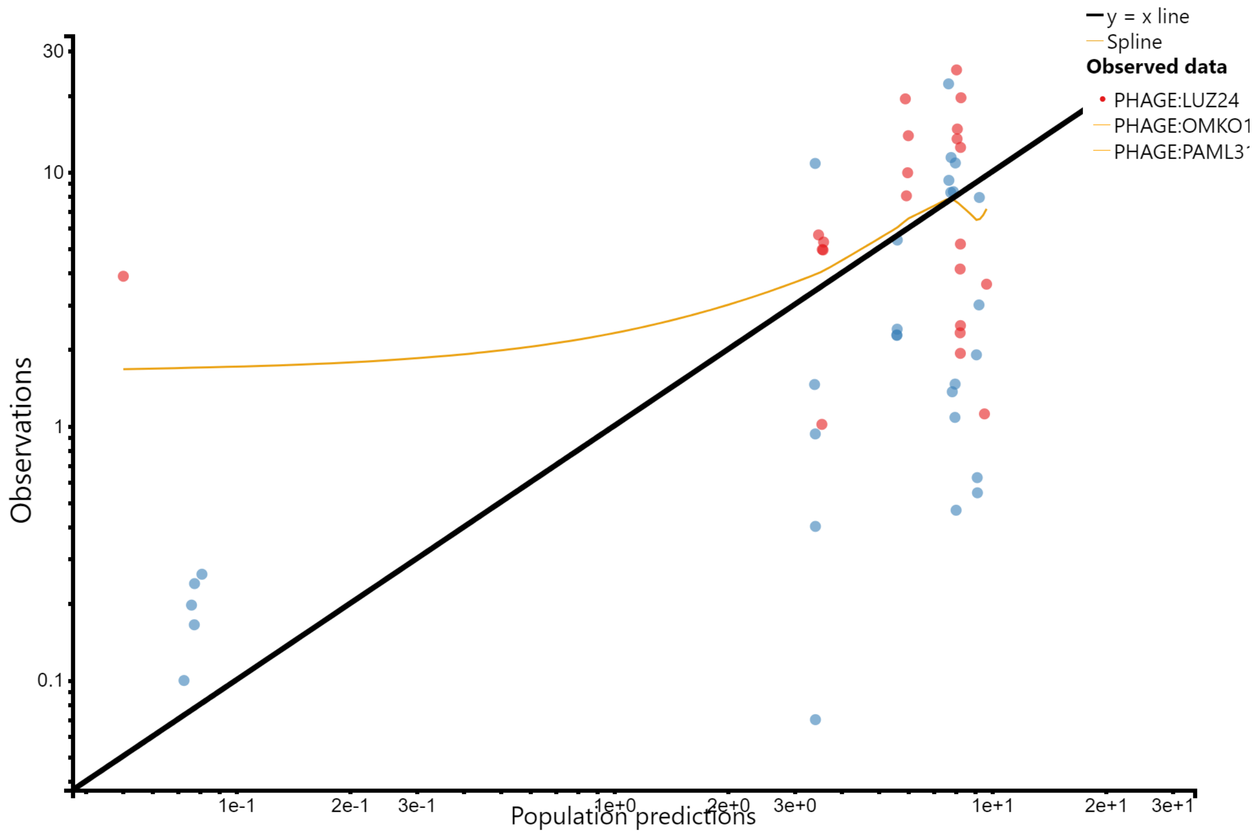

bioRxiv preprint doi: https://doi.org/10.1101/2025.02.06.636931; this version posted February 6, 2025. The copyright holder for this preprint (which was not certified by peer review) is the author/funder, who has granted bioRxiv a license to display the preprint in perpetuity. It is made available under aCC-BY 4.0 International license.

Figure S13: Global Sensitivity Analysis (GSA) of key model parameters

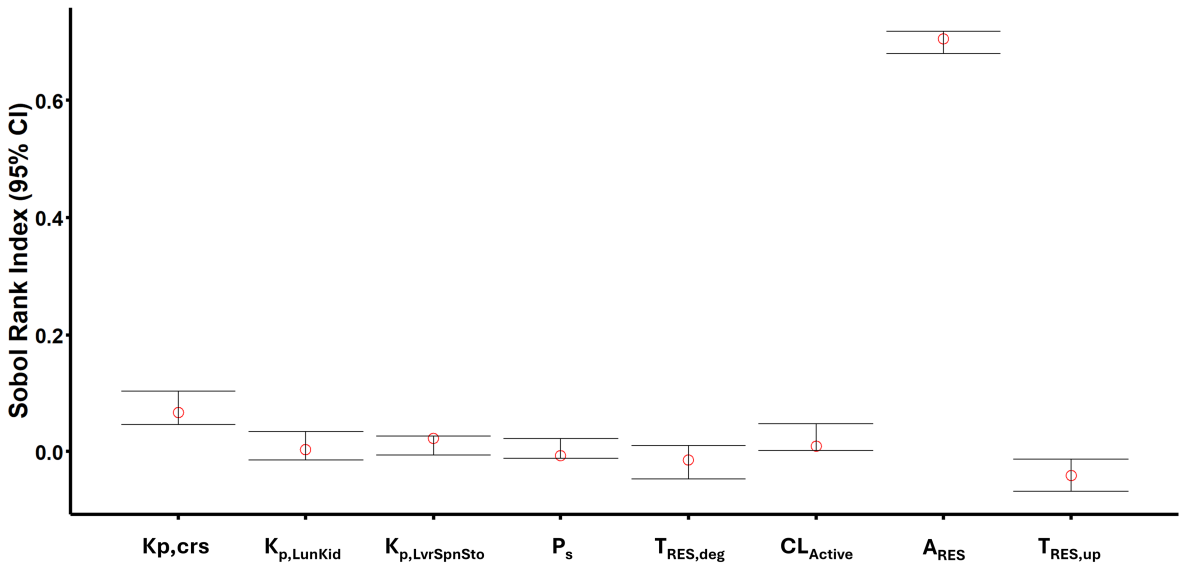

bioRxiv preprint doi: https://doi.org/10.1101/2025.02.06.636931; this version posted February 6, 2025. The copyright holder for this preprint (which was not certified by peer review) is the author/funder, who has granted bioRxiv a license to display the preprint in perpetuity. It is made available under aCC-BY 4.0 International license.

Table S2: Global Sensitivity Analysis (GSA) of Key Model Parameters

First order rank Sobol indices for each parameter based on 1000 simulated parameter sets and 95% confidence intervals.

	First-order rank	bias	std. error	min. c.i.	max. c.i.
$K_{p,Crs}$	0.066631	-0.00678	0.020091	0.045669	0.102709
$K_{p,\ LunKid}$	0.003565	-0.00394	0.016095	-0.01503	0.034368
$K_{p,\ LvrSpnSto}$	0.021637	0.006517	0.010788	-0.00516	0.026982
P_S	-0.00742	-0.01114	0.010871	-0.01188	0.021841
$T_{MPS,\ Deg}$	-0.01457	0.003462	0.019097	-0.04674	0.009832
CL_{Active}	0.009487	-0.00908	0.016183	0.002325	0.046665
A_{MPS}	0.704755	0.001264	0.010696	0.679477	0.719241
$T_{MPS,\ Up}$	-0.04068	0.001177	0.01736	-0.06836	-0.01349

bioRxiv preprint doi: https://doi.org/10.1101/2025.02.06.636931; this version posted February 6, 2025. The copyright holder for this preprint (which was not certified by peer review) is the author/funder, who has granted bioRxiv a license to display the preprint in perpetuity. It is made available under aCC-BY 4.0 International license.

Figure S14: PBPK Post Hoc fits, first 8 hours

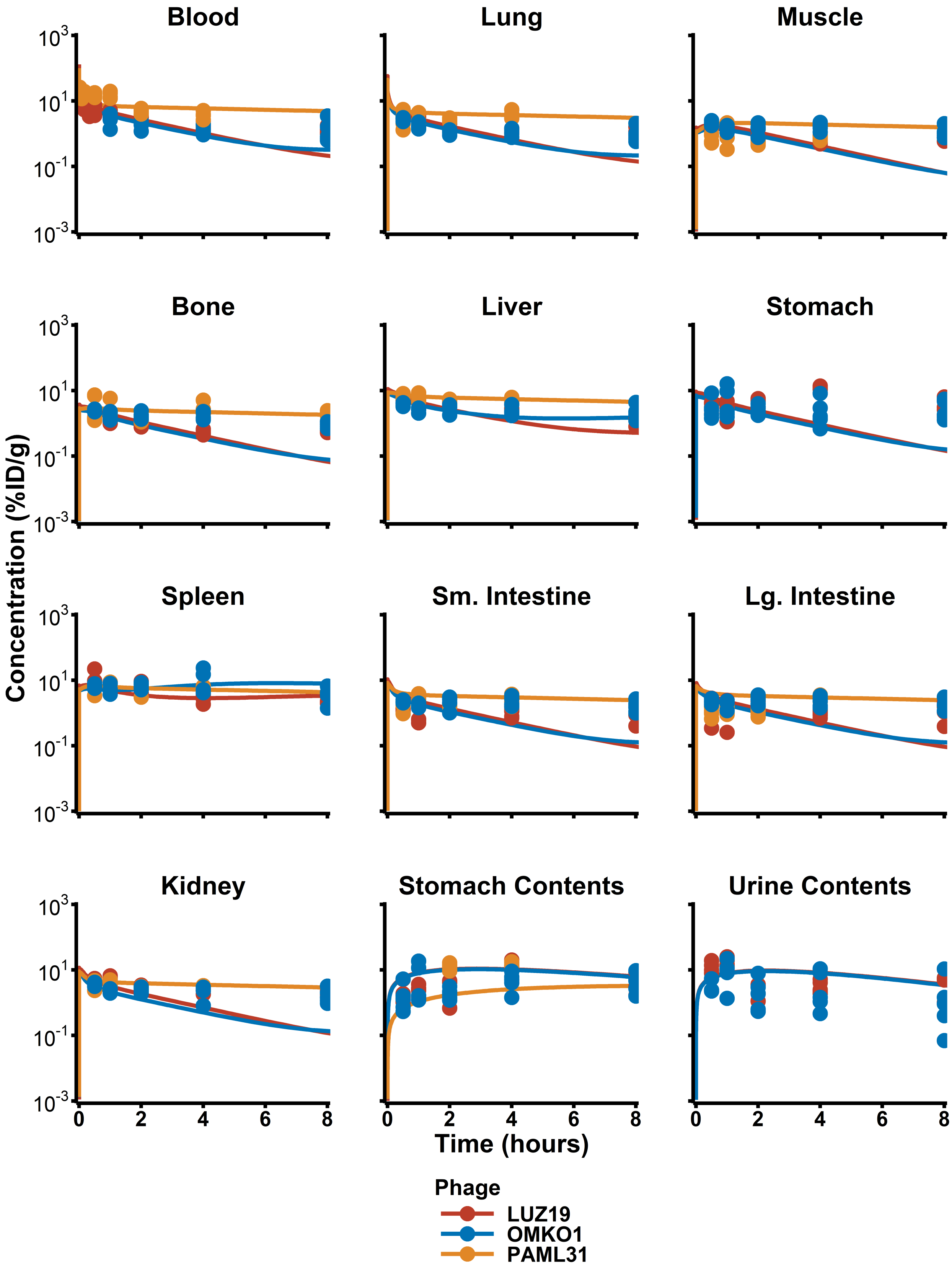

Supplement: 1 [file NIHPP2025.02.06.636931v1-supplement-1.pdf]
